# Supplementary material for: Modelling Skylarks (Alauda arvensis) to Predict Impacts of Changes in Land Management and Policy: Development and Testing of an Agent-Based Model
Source: PLoS One. 2013 Jun 6;8(6):e65803. doi: 10.1371/journal.pone.0065803 (PMC3675089; doi:10.1371/journal.pone.0065803)
Supplement: Supporting Information S4 — The skylark ODdox as a zipped archive. (ZIP) [file pone.0065803.s004.zip › Skylark_ODdox/dir_fac5040e2291d2cdebf3369e2596937b.html]

ALMaSS Skylark ODdox: C:/CJT/MSVC/ALMaSS Working Source/Landscape/ Directory Reference


ALMaSS Skylark ODdox
 2.0


- Main Page
- Related Pages
- Classes
- Files

Landscape Directory Reference

|  |  |
| --- | --- |
| Files | |
| file | calendar.cpp |
| file | calendar.h |
| file | CIPELandscapeMaker.h |
| file | configurator.cpp |
| file | configurator.h |
| file | croprotation.cpp |
| file | croprotation.h |
| file | daylength.h |
| file | elements.cpp |
| file | elements.h |
| file | farm.cpp |
|  | **Farm.cpp This file contains the source for the Farm class** |
| file | farm.h |
|  | **Farm.h This file contains the headers for the Farm class** |
| file | farmfuncs.cpp |
|  | **Farmfuncs.cpp This file contains the source for implementing the farm events** |
| file | hedgebanks.cpp |
| file | Landscape.cpp |
| file | landscape.h |
| file | lowqueue.h |
|  | **lowqueue.h This file contains the headers for the farm event queue classes** |
| file | ls.h |
| file | map\_cfg.cpp |
| file | map\_cfg.h |
| file | maperrormsg.cpp |
| file | maperrormsg.h |
| file | misc.cpp |
| file | pesticide.cpp |
| file | pesticide.h |
| file | plants.cpp |
| file | plants.h |
| file | rastermap.cpp |
| file | rastermap.h |
| file | Rodenticide.cpp |
| file | Rodenticide.h |
| file | tole\_declaration.h |
| file | tov\_declaration.h |
| file | treatment.h |
| file | weather.cpp |
| file | weather.h |


- CJT
- MSVC
- ALMaSS Working Source
- Landscape
- Generated on Thu Jan 10 2013 13:15:38 for ALMaSS Skylark ODdox by
   1.8.1.1
